# The α-Helical Amphipathic Peptide Alleviates Colistin-Induced Nephrotoxicity by Maintaining Mitochondrial Function in Both In Vitro and In Vivo Infection Models

**DOI:** 10.3390/antibiotics14050445

**Published:** 2025-04-28

**Authors:** Min Soo Kook, Heeseung Kim, Yoonhwa Choi, Seong Man Bae, Jaehoon Yu, Yang Soo Kim

**Affiliations:** 1 Department of Biomedical Sciences, University of Ulsan College of Medicine, Seoul 05505, Republic of Korea; maverickoo@amc.seoul.kr; 2 Center for Antimicrobial Resistance and Microbial Genetics, University of Ulsan College of Medicine, Seoul 05505, Republic of Korea; hskim0717@amc.seoul.kr (H.K.); songman.b@amc.seoul.kr (S.M.B.); 3 Division of Infectious Diseases, Department of Internal Medicine, Asan Medical Center, University of Ulsan College of Medicine, Seoul 05505, Republic of Korea; 4 CAMP Therapeutics, Seoul 02139, Republic of Korea; mycat@snu.ac.kr (Y.C.); jhoonyu@snu.ac.kr (J.Y.); 5 Department of Chemistry and Education, Seoul National University, Seoul 08826, Republic of Korea

**Keywords:** CMP3029, α-helical amphipathic peptide, colistin, nephrotoxicity, reactive oxygen species

## Abstract

**Background/Objective**: Colistin is the primary treatment for carbapenem-resistant Gram-negative bacteria (CR-GNB) infections, but its use is limited by nephrotoxicity, which reduces its effectiveness. There is an urgent need for nephroprotective agents to address this toxicity. This study investigated the potential of CMP3029, an α-helical peptide, to protect against colistin-induced nephrotoxicity. **Methods**: In vitro, CMP3029 was applied to HK-2 cells before colistin exposure, and cell viability and reactive oxygen species (ROS) levels were measured. In infected mice, CMP3029 was administered before colistin treatment, and urinary kidney injury molecule-1 (KIM-1), cystatin C levels, neutrophil gelatinase-associated lipocalin (NGAL), and renal damage were assessed. **Results**: CMP3029 preserved cell viability and significantly reduced mitochondrial ROS in HK-2 cells exposed to colistin. CMP3029 lowered urinary biomarkers and mitigated tubular injury in mice, demonstrating significant nephroprotective effects. **Conclusions**: These findings suggest that CMP3029 mitigates colistin-induced nephrotoxicity. Given the increasing threat of CR-GNB infections, CMP3029 could be a crucial clinical solution for improving patient outcomes in treating colistin-associated nephrotoxicity.

## 1. Introduction

Carbapenem-resistant Gram-negative bacteria (CR-GNB) were classified as a critical priority for research and new antibiotic development by the World Health Organization in 2017 [1]. Colistin, or polymyxin E, is one of the few effective options against CR-GNB infections despite its known nephrotoxicity [2]. Developing nephroprotective drugs is crucial for treating CR-GNB infections with colistin, but the overall effectiveness of these agents has not been thoroughly studied [3]. Recent research has focused on oxidative stress, identified as a major factor in colistin-induced kidney toxicity. This toxicity is triggered by mitochondria-generated reactive oxygen species (ROS) that induce kidney cell apoptosis, leading to kidney dysfunction [4,5].

Cell-penetrating peptides (CPPs) can traverse cellular barriers to facilitate transport and exhibit their biological activities, making them promising candidates for disease treatment [6,7]. Recent studies investigated the α-helical amphipathic peptide for its binding affinity to cardiolipin in mitochondria, demonstrating preservation of the cristae structure in the mitochondrial membrane in both in vitro and in vivo models [8]. Building on this research, our study examined CMP3029, an α-helical amphipathic peptide hypothesized to support mitochondrial function during colistin treatment for infections. The nephroprotective effects of CMP3029 were evaluated using an in vitro HK-2 cell model and an in vivo mouse thigh infection model.

## 2. Results

### 2.1. CMP3029 Improved the Cell Viability of the HK-2 Cell Line When Exposed to Colistin

**Figure 1:**
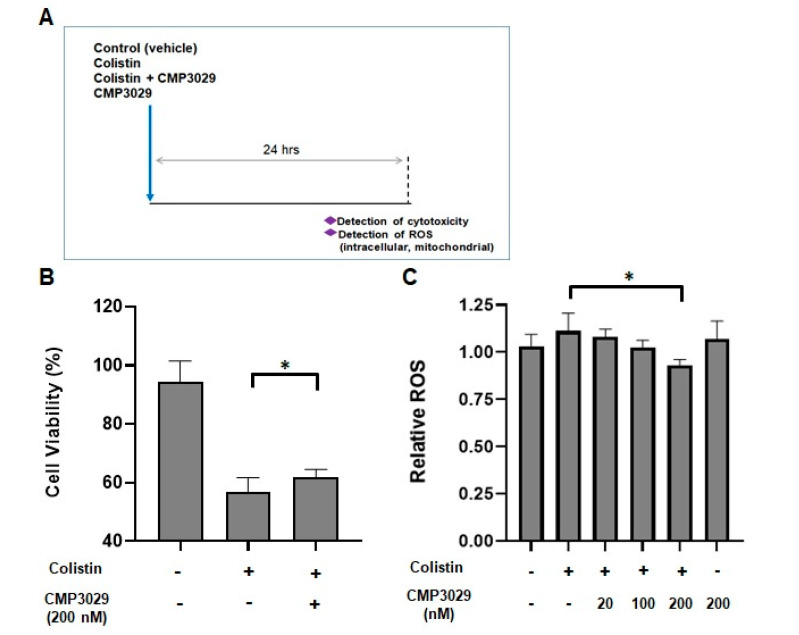

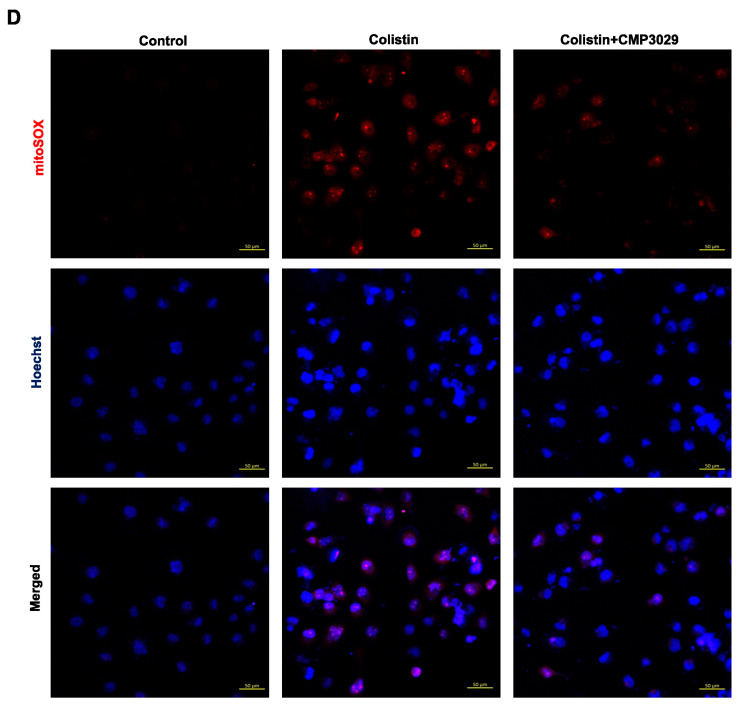
Cell viability, intracellular ROS, and mitochondrial ROS levels in HK-2 cells treated with colistin and/or CMP3029: (**A**) An experimental scheme for an in vitro system using a human proximal tubule cell (HK-2 cell) was used. (**B**) The viability of HK-2 cells treated with colistin with/without CMP3029 for 24 h. (**C**) Intracellular ROS production of HK-2 cells measured by 2′,7′-dichlorofluorescein diacetate (DCFDA). (**D**) Mitochondrial ROS production of HK-2 cells measured by MitoSOX. Data represent means ± SEM and represent three replicate assays per group. * *p* < 0.05 at each group (×200 magnification).

After 24 h of exposure to colistin (400 μg/mL), only 56.74 ± 4.97% of HK-2 cells remained viable (Figure 1B). However, co-treatment with CMP3029 (200 nM) and colistin preserved 61.87 ± 2.62% of the cells (Figure 1B). This dosage of colistin and CMP3029 was selected for further study.

### 2.2. CMP3029 Attenuated Colistin-Induced Intracellular and Mitochondrial ROS Overproduction of HK-2 Cells

Intracellular ROS levels increased in HK-2 cells exposed to colistin alone (400 μg/mL) for 24 h but were significantly reduced when treated with CMP3029 (Figure 1C). Mitochondrial ROS levels also decreased to 83.7% in the presence of CMP3029 (Figure 1D).

### 2.3. CMP3029 Mitigated Colistin-Induced Nephrotoxicity in an In Vivo Infection Model

**Figure 2:**
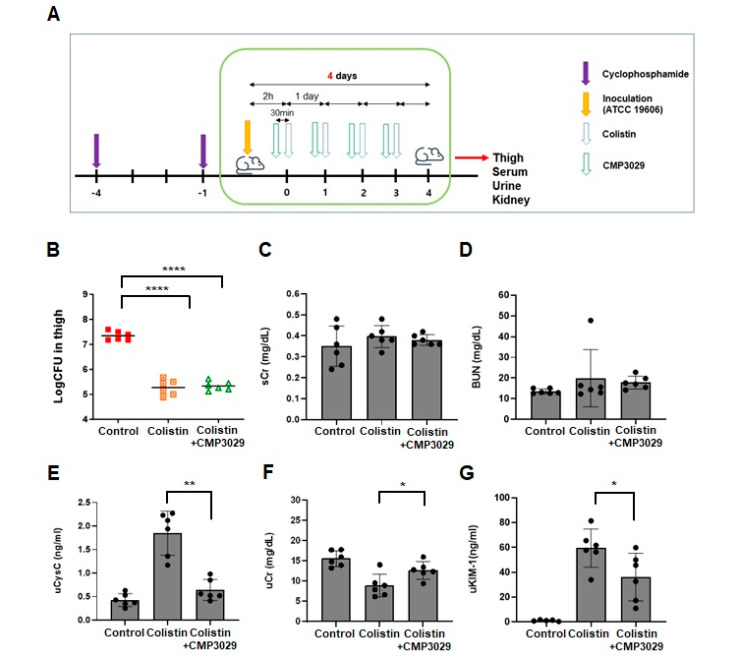

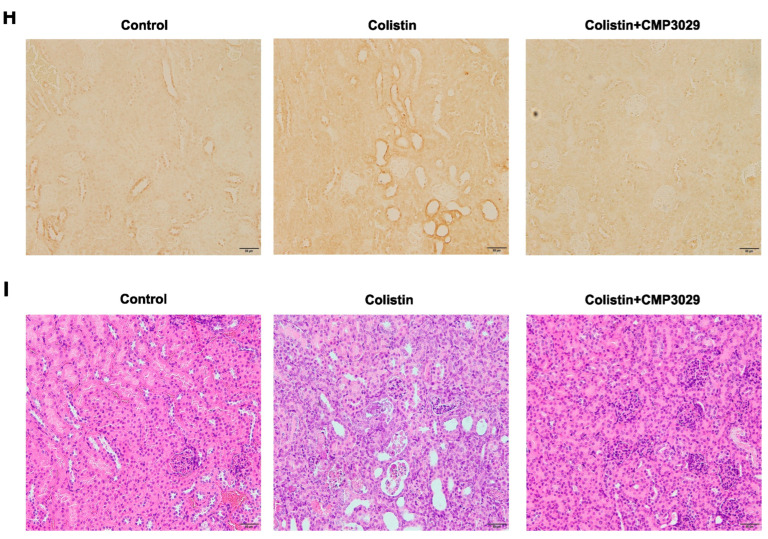
Nephroprotective effect of CMP3029 in *A. baumannii*-infected mice treated with colistin. (**A**) Experimental scheme for the in vivo system (on mice). (**B**–**I**) Female C57BL/6 mice were injected with ATCC29213 (2 × 10^7^ cfu/thigh) on the right thigh of each mouse. Colistin was administered at 25 mg/kg (mpk) once daily for 4 consecutive days. (n = 6 for each group) (**B**) Colony counting in thigh homogenate. (**C**,**D**) BUN, Cr in serum. (**E**–**G**) Cr, cystatin C, and KIM-1 in urine. (**H**) Immunohistochemistry examination to NGAL. (**I**) Hematoxylin and eosin staining (×200 magnification) * *p* < 0.05, ** *p* < 0.01, *** *p* < 0.001, **** *p* < 0.0001 at each group.

Based on the in vitro results, the nephroprotective effects of CMP3029 were investigated in a neutropenic thigh infection model. After *A. baumannii* infection of the thigh and subsequent treatment with colistin or CMP3029, colony-forming units (CFU) in the thigh were log 7.3 ± 0.18 in the saline-treated group. In comparison, CFU levels were log 5.2 ± 0.31 (*p* < 0.0001) with colistin alone and 5.3 ± 0.18 (*p* < 0.0001) with the combined colistin and CMP3029 treatment. Similarly, following *K. pneumoniae* infection, CFU levels were log 7.5 ± 0.14 in the saline-treated group, reduced to log 5.4 ± 0.12 (*p* < 0.0001), with colistin alone, and further decreased to log 5.39 ± 0.25 (*p* < 0.0001) with the combined treatment. Four days after *A. baumannii* infection, serum BUN and Cr levels were measured across the groups. In the colistin-only group, serum BUN and Cr levels were 13.6 ± 0.05 mg/dL and 0.35 ± 0.1 mg/dL, respectively (Figure 2B,C).

In contrast, slightly elevated levels of 17.83 ± 3.09 mg/dL and 0.38 ± 0.03 mg/dL were observed with the combination treatment of colistin and CMP3029 (all *p* > 0.05) (Figure 2B,C). However, uCr levels significantly decreased to 8.86 ± 2.85 mg/dL in the colistin-only group compared to the control group (12.67 ± 2.13 mg/dL) (Figure 2F). Notably, pretreatment with CMP3029 restored uCr levels to 12.67 ± 2.13 mg/dL, similar to the control group (15.58 ± 1.92 mg/dL). (Figure 2E). Urinary KIM-1 levels were reduced to 36.09 ± 19.22 ng/mL compared to 59.48 ± 15.42 ng/mL in the control group, while urinary cystatin C levels decreased to 0.64 ± 0.22 ng/mL relative to 1.85 ± 0.47 ng/mL in the control group (Figure 2F,G). 

**Figure A1:**
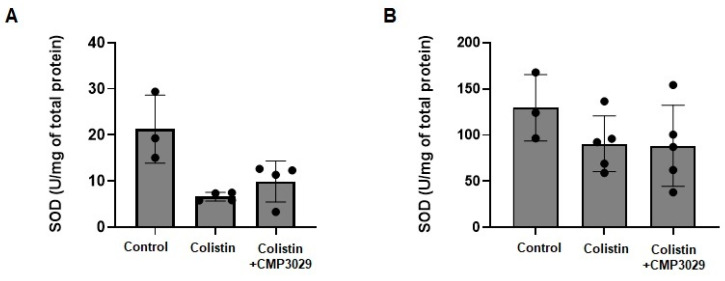
Impact of CMP3029 supplementation on the levels of superoxide dismutase (SOD) in the kidney tissues of mice treated with colistin: (**A**) SOD activity in the tissues of mice infected with ATCC 19606 and treated with colistin or CMP3029 for 4 consecutive days. (n = 3 or 4 on each group). (**B**) SOD activity in the tissues of mice infected with ATCC 700603 and treated with colistin or CMP3029 for 4 consecutive days (n = 3 or 5 in each group).

Furthermore, the CMP3029-treated group showed decreased levels of NGAL (Figure 2H). In the kidneys of the colistin group, acute damage with tubular dilation and pale tubular casts was observed, but not in the other groups (Figure 2I). SOD activity was significantly decreased to 6.62 ± 6.01 U/mg protein by colistin treatment. In contrast, CMP3029 supplementation exhibited a restoring tendency to 9.91 ± 3.83 U/mg protein (*p* = 0.195) (Appendix A Figure A1A). 

**Figure 3:**
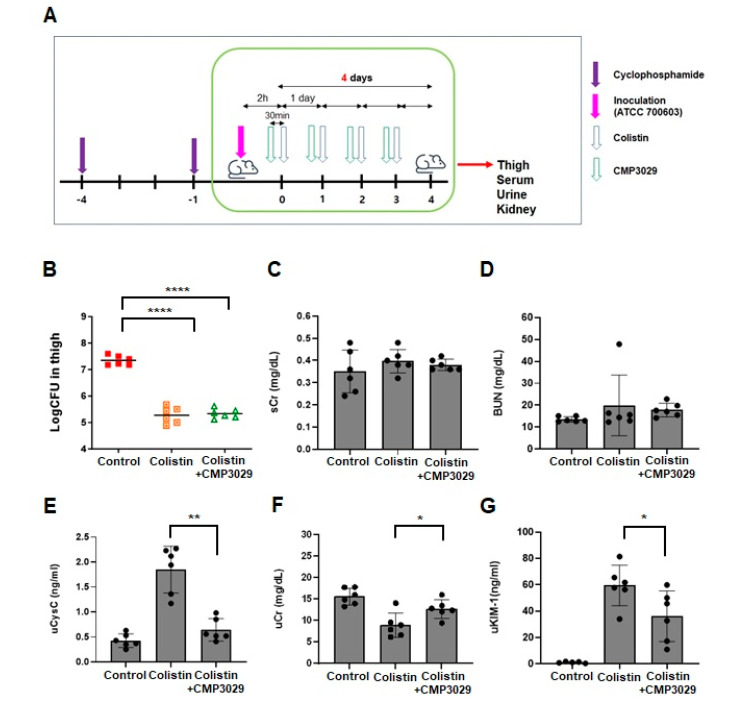

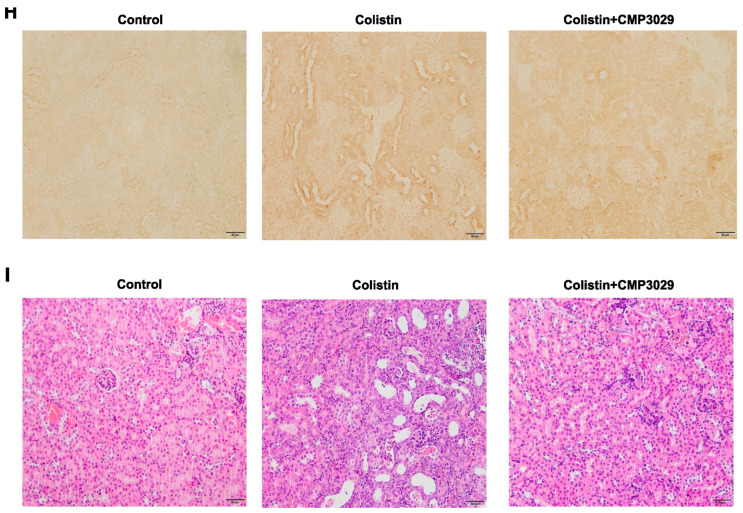
Nephroprotective effect of CMP3029 in *K. pneumoniae*-infected mice treated with colistin. (**A**) Experimental scheme for the in vivo system (on mice). (**B**–**I**) Female C57BL/6 mice were injected with ATCC 700603 (1.8 × 10^7^ cfu/thigh) on the right thigh of each mouse. Colistin was administered at 25 mg/kg (mpk) once daily for 4 consecutive days. (n = 3 or 5 for each group) (**B**) Colony counting in thigh homogenate. (**C**,**D**) BUN, Cr in serum. (**E**–**G**) Cr, cystatin C, and KIM-1 in urine. (**H**) Immunohistochemistry examination to NGAL. (**I**) Hematoxylin and eosin staining (x200 magnification) * *p* < 0.05, ** *p* < 0.01, *** *p* < 0.001, **** *p* < 0.0001 at each group.

Similarly, following *K. pneumoniae* infection, serum BUN and Cr levels were assessed across treatment groups. In the colistin-only group, these levels were 21.32 ± 7.76 mg/dL and 0.37 ± 0.07 mg/dL, respectively (Figure 3B,C). Slightly lower levels of 19.34 ± 8.07 mg/dL and 0.34 ± 0.05 mg/dL were observed in the group receiving the combination treatment of colistin and CMP3029 (all *p* > 0.05) (Figure 3B,C). UCr levels decreased to 9.31 ± 2.94 mg/dL (*p* > 0.05) in the colistin-only group compared to the control group, but were restored to 12.69 ± 2.37 mg/dL by pretreatment with CMP3029, approaching the control group value (15.65 ± 2.28 mg/dL) (Figure 3E). Furthermore, urinary KIM-1 levels decreased to 67 ± 31.14 ng/mL in the combination treatment group compared to 220 ± 117.7 ng/mL in the control group. Urinary cystatin C levels also significantly decreased to 606 ± 148.09 ng/mL (*p* < 0.001) compared to 1.3 ± 0.27 μg/mL in the control group (Figure 3F,G). The CMP3029-treated group exhibited a reduction in NGAL levels (Figure 3H). In the colistin group, kidney damage included tubular dilation and pale tubular casts, which were not observed in the other groups (Figure 3I). SOD activity was reduced to 89.28 ± 29.88 U/mg protein by colistin treatment. CMP3029 supplementation showed the activity to 101.07 ± 33.63 U/mg protein (*p* = 0.93) (Appendix A Figure A1B).

## 3. Discussion

Patients receiving colistin treatment face a critical challenge due to the necessity of higher doses for adequate bacterial clearance. This toxicity is primarily attributed to the accumulation of colistin within renal cells [9]. Antioxidants such as ascorbic acid and curcumin have been explored as nephroprotective agents, but their clinical application has been limited by the high doses required and the limited efficacy observed in clinical trials [10,11]. While CMP3029 is not a classical antioxidant, it may confer mitochondrial protection indirectly by modulating oxidative stress pathways. These findings support continued efforts to develop agents that mitigate ROS-related injury and improve the safety profile of colistin therapy.

This study used an infection model to administer colistin at nephrotoxic doses while treating the infection, allowing for a more clinically relevant assessment compared to traditional models using normal mice [10,12]. Gradually increasing colistin dosages were administered to prevent initial manifestations of overt neurotoxicity associated with higher starting doses [13]. Recent research suggests that changes in serum creatinine may not indicate kidney damage. KIM-1 and cystatin C were analyzed as biomarkers for detecting subclinical acute kidney injury (AKI) before serum creatinine levels rise, helping prevent irreversible kidney damage [14,15]. NGAL was also chosen as a critical indicator of nephrotoxicity and is increasingly used to monitor nephrotoxicity in the early stage [16]. Urine analysis was selected as a non-invasive and highly sensitive method for diagnosing nephrotoxicity, offering earlier detection compared to sCr analysis, as supported by previous studies [17].

**Figure A2:**
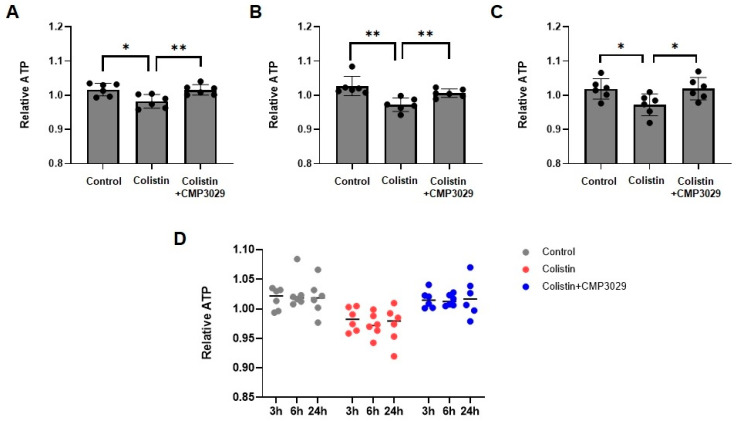
Impact of CMP3029 supplementation on the levels of ATP in the HK-2 cell treated with colistin and/or CMP3029: (**A**) ATP production in the HK-2 cell treated with colistin or CMP3029 for 3 h. (**B**) ATP production in the HK-2 cell treated with colistin or CMP3029 for 6 h. (**C**) ATP production in the HK-2 cell treated with colistin or CMP3029 for 24 h. (**D**) ATP production in HK-2 cells treated with colistin or CMP3029 over time. Data represent means ± SEM and represent six replicate assays per group. * *p* < 0.05, ** *p* < 0.01 at each group.

Unlike curcumin and ascorbic acid, which are known non-enzymatic ROS scavengers requiring doses of at least 150 mg/kg for efficacy, CMP3029 is effective at nanomolar concentrations (200 nM) in vitro and at 1 mg/kg in infected mice in vivo. A previous study on CMP3013, an α-helix dimeric peptide differing by four hydrophobic amino acids, tested its ability to mitigate oxidative stress in human umbilical vein endothelial cells (HUVECs) cell lines using hydrogen peroxide and normal mice. The study demonstrated CMP3013’s organelle specificity in binding mitochondrial cardiolipin and preventing pathological remodeling of the IMM [8]. While direct visualization of mitochondrial structural preservation by CMP3029 has not yet been performed, functional readouts such as preserved ATP production under colistin exposure (Appendix A Figure A2) suggest its potential to support mitochondrial integrity. These findings support the hypothesis that CMP3029 may mitigate colistin-induced nephrotoxicity by maintaining mitochondrial function, even in the absence of confirmed structural data.

CMP3029’s protective effect may be associated with reduced mitochondrial oxidative stress, which is recognized as a key factor in colistin-induced nephrotoxicity. In vitro, CMP3029 has been shown to reduce mitochondrial ROS overproduction, helping preserve cellular viability at 200 nM CMP3029 (Figure 1D). These findings are consistent with previous reports [10,12] implicating oxidative damage in colistin-induced kidney injury. While the exact mechanism remains to be fully elucidated, our results support the potential of CMP3029 as a nephroprotective agent that may mitigate mitochondrial damage and oxidative stress during colistin therapy.

The nephroprotective potential of CMP3029 has been significantly supported by in vivo studies. Bacterial clearance in the thigh infection model indicated that colistin’s therapeutic efficacy was unaffected (Figure 2A and Figure 3A). Significant reductions in nephrotoxicity biomarkers, including urinary KIM-1 and cystatin C, were observed without notable changes in serum creatinine levels. Histological analysis showed reduced NGAL accumulation and kidney damage in the CMP3029-pretreated group (Figure 2 and Figure 3). Notably, CMP3029 protected at doses as low as 1 mg/kg, while antioxidants like curcumin and ascorbic acid require doses of 150 or 200 mg/kg for preclinical efficacy [10,18]. CMP3029’s efficacy at low doses highlights its potential clinical advantages.

The significant reductions in urinary biomarkers and histological evidence of reduced NGAL accumulation and kidney damage further substantiate CMP3029’s nephroprotective potential. These findings, combined with its low-dose efficacy, demonstrate CMP3029’s promising clinical advantages in preventing colistin-induced nephrotoxicity. Significant histological abnormalities and larger affected regions were observed in the kidneys of the colistin-only group. In contrast, less damage and smaller affected regions were noted in the colistin and CMP3029-pretreated group (Figure 2 and Figure 3, and Appendix A Table A1 and Table A2). These findings strongly support CMP3029’s nephroprotective potential against colistin-induced kidney injury.

The specific mechanism by which CMP3029 attenuates colistin-induced nephrotoxicity is unknown. To identify this mechanism, it has been reported that colistin accumulation in proximal tubule cells reduces superoxide dismutase (SOD) activity, which is crucial for neutralizing superoxide radicals and considered a promising target for preventing kidney disease [6,10]. Notably, SOD activity restoration in the kidneys was observed in 75% of the mice in the colistin and pretreated CMP3029 group, compared to the colistin-only treated group infected with *A. baumannii* (*p* > 0.05) (Appendix A Figure A1). However, for SOD activity in the kidneys of mice infected with *K. pneumoniae*, no significant difference was observed between the two groups (*p* > 0.05) (Appendix A Figure A1B). However, CMP3029 demonstrates potent nephroprotection, effectively reducing colistin-induced oxidative stress in vitro. This trend suggests that CMP3029 may modulate oxidative stress through mechanisms beyond classical antioxidant activity. The consistency between in vitro and in vivo efficacy highlights its therapeutic potential, reinforcing its relevance despite the inherent variability in antioxidant responses.

Further studies are needed to clarify the mechanisms by which CMP3029 influences mitochondrial function and modulates ROS production in human cells. Although direct interaction between CMP3029 and mitochondrial structures has not yet been demonstrated, preserved ATP production under colistin exposure with CMP3029 Appendix A Figure A2) suggests a potential protective effect on mitochondrial function. Clinical trials will be essential to evaluate the safety and efficacy of CMP3029 in patients receiving colistin therapy, and to determine whether CMP3029 provides therapeutic advantages over traditional antioxidants, which typically require higher doses for comparable efficacy. It also remains important to investigate whether CMP3029 directly affects bacterial viability or pathogen clearance, as these aspects have not yet been explored.

While CMP3029 demonstrated significant mitigation of mitochondrial ROS overproduction induced by colistin in vitro, its effects were primarily observed at the cellular and organ levels, rather than directly influencing bacterial viability. In our infected thigh model, no significant difference in colony-forming unit (CFU) counts was detected between the colistin-only group and the colistin and CMP3029-pretreated group (Figure 2 and Figure 3). This indicates that CMP3029 likely acts on host-cell physiological mechanisms rather than exhibiting direct antimicrobial activity. We acknowledge this limitation and propose further investigation to determine whether CMP3029 may impact immune responses or other host–pathogen interaction pathways.

Accurately measuring mitochondrial ROS in renal tubular cells presents several challenges [19,20]. General ROS dyes often lack specificity, making it difficult to distinguish mitochondrial from cytoplasmic signals. Mitochondrial-targeted probes like MitoSOX require live-cell imaging and are influenced by membrane potential and dye uptake variability. Additionally, excessive ROS during sample preparation can create artifacts, complicating interpretation. These limitations justify the use of complementary indicators, such as ATP levels, to indirectly assess mitochondrial oxidative stress and function.

Despite these technical constraints, our data provide compelling functional evidence that CMP3029 mitigates mitochondrial dysfunction. The observed correlation between reduced intracellular and mitochondrial ROS (Figure 1B,C) and preserved ATP production (Appendix A Figure A2) suggests that CMP3029 helps maintain mitochondrial integrity under colistin-induced stress. While direct structural confirmation is pending, this functional consistency strengthens the argument that CMP3029 protects renal tubular cells by modulating mitochondrial oxidative stress. Given the limitations of current ROS detection techniques, the preservation of cellular energy metabolism offers a robust and physiologically relevant indicator of mitochondrial health.

Targeting and modulating ROS has emerged as a validated therapeutic approach for mitigating nephrotoxicity. Numerous studies have demonstrated that antioxidants such as curcumin, ascorbic acid, and N-acetylcysteine (NAC) confer protective effects in various models of kidney injury by scavenging free radicals and enhancing endogenous antioxidant responses, particularly through activation of the Nrf2 signaling pathway [21,22]. Genetic interventions have also been explored; overexpression of mitochondrial antioxidant enzymes such as superoxide dismutase 2 (SOD2) has been shown to reduce mitochondrial oxidative damage and attenuate renal dysfunction in ischemia–reperfusion and toxin-induced injury models [23]. Moreover, mitochondria-targeted therapeutic peptides like SS-31 have proven effective in directly suppressing mitochondrial ROS and preventing tubular epithelial cell apoptosis [24]. These studies provide a robust scientific framework supporting the therapeutic rationale for CMP3029, which appears to modulate oxidative stress and preserve mitochondrial function under colistin-induced nephrotoxicity.

CMP3029 demonstrates significant nephroprotective effects against colistin-induced nephrotoxicity, showing efficacy at much lower doses compared to traditional antioxidants. A major strength of this study is the ability of CMP3029 to maintain mitochondrial function. First, CMP3029 effectively reduces mitochondrial ROS overproduction. Second, functional indicators such as preserved ATP production support the interpretation of CMP3029’s role in protecting mitochondrial integrity. These actions not only minimize nephrotoxic risk but also potentially expand the therapeutic window of colistin, making its use safer for patients. These findings highlight CMP3029 as a promising candidate for mitigating colistin-induced nephrotoxicity. Moreover, the study underscores the potential of CMP3029 as a low-dose alternative to conventional antioxidants, which often require high concentrations to achieve similar therapeutic effects. Nonetheless, further research is needed to fully elucidate its mechanisms of action and to confirm its safety and efficacy in clinical settings.

## 4. Materials and Methods

### 4.1. Chemicals

Colistin sulfate salt and cyclophosphamide were purchased from Sigma-Aldrich. CMP3029 was generously provided by CAMP Therapeutics Co., Ltd. (CAMP Therapeutics, Seoul, Republic of Korea).

### 4.2. Cell Culture

HK-2 cells (Human renal proximal tubular epithelial cell line) were obtained from the Korean Cell Line Bank (the Korean Cell Line Bank, Seoul, Republic of Korea). These cells were cultured in DMEM/F12 medium supplemented with 10% fetal bovine serum (FBS) and 1% penicillin/streptomycin. Cells were incubated at 37 °C under 5% CO_2_ in a humidified incubator.

### 4.3. Mice

Female C57BL/6N mice (8~9 weeks old) were purchased from JABIO Co., Ltd. (JABIO, Goyang-si, Republic of Korea). Mice were housed in controlled, specific pathogen-free environments and subjected to a 12 h light/dark cycle. All experimental procedures were approved by the Institutional Animal Care and Use Committee of the Asan Institute for Life Sciences (2022-12-316).

### 4.4. Bacterial Strains

*Acinetobacter baumannii* ATCC 19606 and *Klebsiella pneumoniae* ATCC 700603 were acquired from the American Type Culture Collection (ATCC, Manassas, VA, USA) for use in this study.

### 4.5. Analysis of Cell Viability

HK-2 cells (1 × 10^4^ cells/well) were seeded into 96-well plates. Cells were treated with or without colistin and CMP3029 for 24 h, and cell viability was measured using MTT reagents (Abcam, Cambridge, UK) according to the manufacturer’s protocol. Absorbance was measured with a microplate spectrophotometer (Molecular Devices Inc., San Jose, CA, USA) at 590 nm.

### 4.6. Measurement of Intracellular ROS Levels

HK-2 cells (1 × 10^4^ cells/well) were seeded into 96-well plates. Cells were incubated and then stained with DCFDA-H_2_DCFDA following the manufacturer’s protocol. The cells were analyzed at 37 °C using a TECAN plate reader (Tecan Trading, Männedorf, Switzerland) with 485 nm excitation and 538 nm emission.

### 4.7. Measurement of Mitochondrial ROS Levels

HK-2 cells were seeded into glass-bottom cell culture dishes (Wuxi NEST Biotechnology, Wuxi, China). The cells were incubated with MitoSOX red dye (ThermoFisher Scientific, Waltham, MA, USA) at 37 °C in 5% CO2. Nuclei were stained with Hoechst 33258 (ThermoFisher Scientific). Fluorescence intensity was observed using a confocal microscope (Zeiss LSM 700, Oberkochen, Germany). Images were processed with ZEN Lite software (Version 3.9.101.03000). All experiments were conducted in triplicate.

### 4.8. Neutropenic Mouse Thigh Infection Model

The experimental methodology followed reference [25]. Four days before infection, each female C57BL/6 mouse (8 weeks old) received a single intraperitoneal injection of 150 mg/kg cyclophosphamide. One day before infection, a 100 mg/kg dose was administered. ATCC 19606 and ATCC 700603 were subcultured on sheep blood agar at 37 °C overnight, then diluted in TSB and grown with shaking at 37 °C. Bacterial cells were centrifuged and resuspended in sterile saline for inoculation. Thigh infection was established by injecting the bacterial suspension into the right thigh of mice (n = 3, 5, or 6). At 1 h 30 min post-infection, mice received a subcutaneous injection of CMP3029 at 1 mg/kg or control solution, one dose every 24 h. At 2 h post-inoculation, Mice received intraperitoneal injections of colistin (25 mg/kg) or an equal volume of saline once daily. At 0 and 108 h post-administration, animals were euthanized, and serum was collected. The right thigh was collected, homogenized under sterile conditions, serially diluted in sterile saline, and spread on Mueller–Hinton agar plates. Agar plates were incubated at 37 °C overnight, and bacterial densities were quantified as log_10_ CFU/thigh.

### 4.9. Urine Collection

Urine was collected using individual conventional metabolic cages from Jeung Do Bio Co., Ltd. (Jeung Do Bio, Paju-si, Republic of Korea), utilizing a modified restraint method for 24 h collection as outlined in reference [26]. Urine samples were centrifuged. The supernatant was stored in 1.5 mL e-tubes at −80 °C.

### 4.10. Determination of Serum and Urinary Biomarkers for Kidney Injury

BUN, serum creatinine (sCr), and urinary creatinine (uCr) were measured using an automatic analyzer (Hitachi 7180) (Hitachi, Tokyo, Japan). Urinary cystatin C and kidney injury molecule-1 (KIM-1) (Abcam, Cambridge, UK) were measured by ELISA.

### 4.11. Immunohistochemistry

Paraffin-embedded samples were cut into 3 μm sections, de-paraffinized, and rehydrated. Slides were incubated in PBS with H_2_O_2_. Samples were incubated with a polyclonal antibody against Lipocalin-2 (LCD2)/neutrophil gelatinase-associated lipocalin (NGAL) (R&D Systems, Minneapolis, MN, USA) overnight at 4 °C. Secondary antibodies were applied for 2 h at room temperature in PBS. Sections were then incubated with DAB substrate solution (Thermo Fisher Scientific), dehydrated, and mounted in Permount^TM^ mounting medium (Fisher Chemical, Waltham, MA, USA).

### 4.12. Histopathological Examination

The left kidney was fixed in 10% neutral buffered formalin for at least 48 h, dehydrated in ethanol, and embedded in paraffin. Kidney tissue blocks were cut into 3 μm sections and stained with hematoxylin–eosin. Coded samples were examined by a pathologist (P. A. H.) who was blinded to the treatment groups. Lesions were graded into three categories: grade 1 (mild acute tubular damage with tubular dilation, prominent nuclei, and a few pale tubular casts); grade 2 (severe acute tubular damage with necrosis of tubular epithelial cells and numerous tubular casts); and grade 3 (acute cortical necrosis/infarction of tubules and glomeruli with or without papillary necrosis). The grades were scored as follows: grade 1 = 1, grade 2 = 4, and grade 3 = 10. The affected kidney slices were scored based on the percentage of damage: <1% = 0, 1% to <5% = 1, 5% to <10% = 2, 10% to <20% = 3, 20% to <30% = 4, 30% to <40% = 5, and ≥40% = 6. The overall score was calculated by multiplying the percentage score and grade score. A semi-quantitative score (SQS) for renal histological changes was assigned as follows: SQS 0 = no significant change (overall score <1), SQS +1 = mild damage (overall score 1 to <15), SQS +2 = mild to moderate damage (overall score 15 to <30), SQS +3 = moderate damage (overall score 30 to <45), SQS +4 = moderate to severe damage (overall score 45 to <60), and SQS +5 = severe damage (overall score 60).

### 4.13. Measurement of Markers of Oxidative Stress in Kidney Tissues

Kidney tissue homogenate was prepared. Cu/Zn-superoxide dismutase (SOD) activity in the homogenate was assessed by commercial kit (Cayman Chemical, Ann Arbor, MI, USA).

### 4.14. Data Analysis

Data were analyzed using the log-rank test. The remaining datasets were evaluated through ANOVAs or t-tests. Post hoc comparisons were performed with the Bonferroni test, with statistical significance set at * *p* < 0.05, ** *p* < 0.01, *** *p* < 0.001, **** *p* < 0.0001 for each group.

## Data Availability

The original contributions presented in this study are included in the article. Further inquiries can be directed to the corresponding author.

## Abbreviations

CR-GNB	Carbapenem-resistant Gram-negative bacteria
ROS	Reactive oxygen species
KIM-1	Kidney injury molecule-1
NGAL	Neutrophil gelatinase-associated lipocalin
CPPs	Cell-penetrating peptides
IMM	Inner mitochondrial membrane
HK-2	Human renal proximal tubular epithelial cells
LCD2	Lipocalin-2
SOD	Superoxide dismutase
BUN	Blood urea nitrogen
sCr	Serum creatinine
uCr	Urinary creatinine
ELISA	Enzyme-linked immunosorbent assay
MTT	3-(4,5-Dimethylthiazol-2-yl)-2,5-diphenyltetrazolium bromide
DCFDA	2′,7′-Dichlorodihydrofluorescein diacetate
SQS	Semi-quantitative score
CFU	Colony-forming unit

## Appendix A

**Table A1:** Histological results for mice infected with *A. baumannii* for 4 days.

	Percentage of the Kidney Slice Affected in Individual Mice of Each Group (n = 5 or 6)			
Abnormality grade	control	colistin	CMP3029	Colistin/CMP3029
1	0, 0, 0, 0, 0	3, 4, 3, 3, 3, 2	0, 0, 0, 0, 0, 0	1, 3, 1, 1, 1, 2
2	0, 0, 0, 0, 0	0, 0, 0, 0, 0, 0	0, 0, 0, 0, 0, 0	0, 0, 0, 0, 0, 0
3	0, 0, 0, 0, 0	0, 0, 0, 0, 0, 0	0, 0, 0, 0, 0, 0	0, 0, 0, 0, 0, 0
SQS for individual mice *	0, 0, 0, 0, 0	1, 1, 1, 1, 1, 1	0, 0, 0, 0, 0, 0	1, 1, 1, 1, 1, 1

SQS *: Semi-Quantitative Score. Grade 1: mild acute tubular damage with tubular dilation, prominent nuclei, and a few pale tubular casts. Grade 2: severe acute tubular damage with necrosis of tubular epithelial cells and numerous tubular casts (acute tubular necrosis). Grade 3: acute cortical necrosis/infarction of tubules and glomeruli with or without papillary necrosis. Colistin/CMP3029 1 mg/kg once daily, respectively. A Semi-Quantitative Score (SQS) was used to grade lesion severity as follows: 0, +1, +2, +3, +4, and +5 correspond to no change, mild, mild to moderate, moderate, moderate to severe, and severe, respectively.

**Table A2:** Histological results for mice infected with *K. pneumoniae* for 4 days.

	Percentage of the Kidney Slice Affected in Individual Mice of Each Group (n = 3 or 5)			
Abnormality grade	control	colistin	CMP3029	Colistin/CMP3029
1	0, 0, 0	3, 3, 3, 4, 2	0, 0, 0, 0, 0, 0	2, 2, 3, 1, 1
2	0, 0, 0	0, 0, 0, 0, 0	0, 0, 0, 0, 0, 0	0, 0, 0, 0, 0
3	0, 0, 0	0, 0, 0, 0, 0	0, 0, 0, 0, 0, 0	0, 0, 0, 0, 0
SQS for individual mice *	0, 0, 0	1, 1, 1, 1, 1	0, 0, 0, 0, 0, 0	1, 1, 1, 1, 1

SQS *: Semi-Quantitative Score. Grade 1: mild acute tubular damage with tubular dilation, prominent nuclei, and a few pale tubular casts. Grade 2: severe acute tubular damage with necrosis of tubular epithelial cells and numerous tubular casts (acute tubular necrosis). Grade 3: acute cortical necrosis/infarction of tubules and glomeruli with or without papillary necrosis. Colistin/CMP3029 1 mg/kg once daily, respectively. An SQS was used to grade lesion severity as follows: 0, +1, +2, +3, +4, and +5 correspond to no change, mild, mild to moderate, moderate, moderate to severe, and severe, respectively.

## References

[1] Moja P.L. (2017). Prioritization of Pathogens to Guide Discovery, Research and Development of New Antibiotics for Drug-Resistant Bacterial Infections, Including Tuberculosis.

[2] Li J., Nation R.L., Milne R.W., Turnidge J.D., Coulthard K. (2005). Evaluation of colistin as an agent against multi-resistant Gram-negative bacteria. Int. J. Antimicrob. Agents.

[3] Mirjalili M., Mirzaei E., Vazin A. (2022). Pharmacological agents for the prevention of colistin-induced nephrotoxicity. Eur. J. Med. Res..

[4] Xie B., Liu Y., Chen C., Velkov T., Tang S., Shen J., Dai C. (2024). Colistin Induces Oxidative Stress and Apoptotic Cell Death through the Activation of the AhR/CYP1A1 Pathway in PC12 Cells. Antioxidants.

[5] Jeong B.Y., Park S.R., Cho S., Yu S.L., Lee H.Y., Park C.G., Kang J., Jung D.Y., Park M.H., Hwang W.M. (2018). TGF-β-mediated NADPH oxidase 4-dependent oxidative stress promotes colistin-induced acute kidney injury. J. Antimicrob. Chemother..

[6] Zhu Y., Luo M., Bai X., Li J., Nie P., Li B., Luo P. (2022). SS-31, a Mitochondria-Targeting Peptide, Ameliorates Kidney Disease. Oxidative Med. Cell. Longev..

[7] Xie J., Bi Y., Zhang H., Dong S., Teng L., Lee R.J., Yang Z. (2020). Cell-Penetrating Peptides in Diagnosis and Treatment of Human Diseases: From Preclinical Research to Clinical Application. Front. Pharmacol..

[8] Shin G., Hyun S., Kim D., Choi Y., Kim K.H., Kim D., Kwon S., Kim Y.S., Yang S.H., Yu J. (2024). Cyclohexylalanine-Containing α-Helical Amphipathic Peptide Targets Cardiolipin, Rescuing Mitochondrial Dysfunction in Kidney Injury. J. Med. Chem..

[9] Dai C., Li J., Tang S., Li J., Xiao X. (2014). Colistin-induced nephrotoxicity in mice involves the mitochondrial, death receptor, and endoplasmic reticulum pathways. Antimicrob. Agents Chemother..

[10] Yousef J.M., Chen G., Hill P.A., Nation R.L., Li J. (2012). Ascorbic acid protects against the nephrotoxicity and apoptosis caused by colistin and affects its pharmacokinetics. J. Antimicrob. Chemother..

[11] Sirijatuphat R., Limmahakhun S., Sirivatanauksorn V., Nation R.L., Li J., Thamlikitkul V. (2015). Preliminary Clinical Study of the Effect of Ascorbic Acid on Colistin-Associated Nephrotoxicity. Antimicrob. Agents Chemother..

[12] Dai C., Tang S., Wang Y., Velkov T., Xiao X. (2017). Baicalein acts as a nephroprotectant that ameliorates colistin-induced nephrotoxicity by activating the antioxidant defence mechanism of the kidneys and down-regulating the inflammatory response. J. Antimicrob. Chemother..

[13] Yousef J.M., Chen G., Hill P.A., Nation R.L., Li J. (2011). Melatonin Attenuates Colistin-Induced Nephrotoxicity in Rats. Antimicrob. Agents Chemother..

[14] van Meer L., Moerland M., Cohen A.F., Burggraaf J. (2014). Urinary kidney biomarkers for early detection of nephrotoxicity in clinical drug development. Br. J. Clin. Pharmacol..

[15] Keirstead N.D., Wagoner M.P., Bentley P., Blais M., Brown C., Cheatham L., Ciaccio P., Dragan Y., Ferguson D., Fikes J. (2014). Early Prediction of Polymyxin-Induced Nephrotoxicity with Next-Generation Urinary Kidney Injury Biomarkers. Toxicol. Sci..

[16] Luo Q.H., Chen M.L., Sun F.J., Chen Z.L., Li M.Y., Zeng W., Gong L., Cheng A.C., Peng X., Fang J. (2014). KIM-1 and NGAL as biomarkers of nephrotoxicity induced by gentamicin in rats. Mol. Cell. Biochem..

[17] Sahre M.D.K., Rogers H. (2015). Biomarker Qualification Program Office of Clinical Pharmacology Full Qualification Package Review.

[18] Mehrab H., Sharifi M., Akhavan A., Aarabi M.-H., Mansourian M., Mosavi E., Moghaddas A. (2023). Curcumin supplementation prevents cisplatin-induced nephrotoxicity: A randomized, double-blinded, and placebo-controlled trial. Res. Pharm. Sci..

[19] Adhikari A., Mondal S., Chatterjee T., Das M., Biswas P., Ghosh R., Darbar S., Alessa H., Althakafy J.T., Sayqal A. (2021). Redox nanomedicine ameliorates chronic kidney disease (CKD) by mitochondrial reconditioning in mice. Commun. Biol..

[20] Murphy M.P., Bayir H., Belousov V., Chang C.J., Davies K.J.A., Davies M.J., Dick T.P., Finkel T., Forman H.J., Janssen-Heininger Y. (2022). Guidelines for measuring reactive oxygen species and oxidative damage in cells and in vivo. Nat. Metab..

[21] Lu M., Yin N., Liu W., Cui X., Chen S., Wang E. (2017). Curcumin Ameliorates Diabetic Nephropathy by Suppressing NLRP3 Inflammasome Signaling. BioMed Res. Int..

[22] Sun L.-N., Liu X.-C., Chen X.-J., Guan G.-J., Liu G. (2016). Curcumin attenuates high glucose-induced podocyte apoptosis by regulating functional connections between caveolin-1 phosphorylation and ROS. Acta Pharmacol. Sin..

[23] Schneider M.P., Sullivan J.C., Wach P.F., Boesen E.I., Yamamoto T., Fukai T., Harrison D.G., Pollock D.M., Pollock J.S. (2010). Protective role of extracellular superoxide dismutase in renal ischemia/reperfusion injury. Kidney Int..

[24] Zhao W.-Y., Han S., Zhang L., Zhu Y.-H., Wang L.-M., Zeng L. (2013). Mitochondria-Targeted Antioxidant Peptide SS31 Prevents Hypoxia/Reoxygenation-Induced Apoptosis by Down-Regulating p66Shc in Renal Tubular Epithelial Cells. Cell. Physiol. Biochem..

[25] Dudhani R.V., Turnidge J.D., Coulthard K., Milne R.W., Rayner C.R., Li J., Nation R.L. (2010). Elucidation of the Pharmacokinetic/Pharmacodynamic Determinant of Colistin Activity against *Pseudomonas aeruginosa* in Murine Thigh and Lung Infection Models. Antimicrob. Agents Chemother..

[26] Kurien B.T., Everds N.E., Scofield R.H. (2004). Experimental animal urine collection: A review. Lab. Anim..

